# Isolation and Characterization of Two New Secondary Metabolites From *Quercus incana* and Their Antidepressant- and Anxiolytic-Like Potential

**DOI:** 10.3389/fphar.2018.00298

**Published:** 2018-04-18

**Authors:** Rizwana Sarwar, Umar Farooq, Sadia Naz, Ajmal Khan, Syed M. Bukhari, Haroon Khan, Nasiara Karim, Imran Khan, Ayaz Ahmed, Ahmed Al-Harrasi

**Affiliations:** ^1^Department of Chemistry, COMSATS Institute of Information Technology Abbottabad, Abbottabad, Pakistan; ^2^UoN Chair of Oman’s Medicinal Plants and Marine Natural Products, University of Nizwa, Nizwa, Oman; ^3^Department of Pharmacy, Abdul Wali Khan University Mardan, Mardan, Pakistan; ^4^Department of Pharmacy, University of Malakand, Chakdara, Pakistan; ^5^Department of Pharmacy, University of Swabi, Swabi, Pakistan; ^6^Dr. Panjwani Center for Molecular Medicine and Drug Research, International Center for Chemical and Biological Sciences, University of Karachi, Karachi, Pakistan

**Keywords:** *Quercus incana*, aromatic acid, anxiolytic- and antidepressant-like effect, diazepam, flumazenil

## Abstract

The ethyl acetate fraction of *Quercus incana* yielded two new compounds [**1** and **2**]. The characterization and structure elucidation of these compounds were carried out through various spectroscopic techniques such as mass spectrometry along with one- and two-dimensional NMR techniques. The structural formula was deduced to be 2-(4-hydroxybutan-2-yl)-5-methoxyphenol [**1**] and 4-hydroxy-3-(hydroxymethyl) pentanoic acid [**2**]. The elevated plus maze (EPM) and light–dark box (LDB) tests (classical mouse models) were performed in order to reveal the anxiolytic potential of both compounds [**1** and **2**]. Both compounds displayed dose-dependent increases in open-arm entries and time spent in open arms in EPM (^∗^*P* < 0.05, ^∗∗^*P* < 0.01), and increased the time spent in the lit compartment and increased transitions between the two compartments in LDB test (^∗^*P* < 0.05, ^∗∗^*P* < 0.01). Co-administration of selective benzodiazepine (BZP) receptor antagonist, flumazenil (2.5 mg/kg) with compounds [**1** and **2**] decreased the anxiolytic-like activity of both compounds in the EPM indicating BZP-binding site of GABA-A receptors are involved in the anxiolytic-like effect. Similarly, both compounds at the dose level of 10 and 30 mg/kg, i.p. exerted pronounced antidepressant-like effect in both forced swimming as well as tail suspension tests (^∗^*P* < 0.05, ^∗∗^*P* < 0.01; ANOVA followed by Dunnett’s *post hoc* test). The effect at 30 mg/kg was comparable to the reference drug imipramine (60 mg/kg).

## Introduction

Stress is known to play a significant part in pathogenesis of mental dysfunctions (Kalueff and Tuohimaa, 2004). Anxiety, a common mental illness, from, which 20% of the adult population is suffering across the globe has become a significant research area in the field of psychopharmacology (Yadav et al., 2008; Sharmen et al., 2014). Anxiety is related to significant disability which may result in negative impacts on the patient’s quality of life (Kudagi et al., 2012). Currently, benzodiazepines (BZPs) are used as the drug of choice for the treatment of several types of anxiety (Gupta et al., 2010). Despite the fact that BZPs are well known for their advantages, their side effects are high, which include sedation, myorelaxation, physical dependence, and anterograde amnesia (Barua et al., 2009).

Depression has been described as the mental disorder, which will become the second biggest global health problem after cardiovascular diseases by 2020 (Reddy, 2010). Depression can appear as comorbid symptom on several psychiatric conditions including response to stress, substance abuse, and chronic diseases including diabetes mellitus, cancer, and hypertension. Depression is also associated with disease causing pain, disability, deformity, and conditions, which may reduce patient’s quality of life and life expectancy. A number of antidepressant drugs are now clinically available, which presumably act via different mechanisms, including the noradrenergic, serotonergic, and/or dopaminergic systems. These drugs include monoamine oxidase inhibitors, tricyclic antidepressant drugs, selective serotonin reuptake inhibitors (SSRIs), and serotonin–norepinephrine reuptake inhibitors (SNRIs) (Gonçalves et al., 2012; Khan et al., 2016). However, these agents have their limitations including inadequate effectiveness over a prolonged period and unwanted side effects (Berton and Nestler, 2006). Recently, there has been a renewed interest in natural compounds, particularly from plants that mitigate anxiety and depressive-like symptoms (González-Trujano et al., 2017).

Plant-derived constituents provide a large source of available pharmaceuticals in modern medicine, which are directly or indirectly derived from natural sources. In drug discovery process, the natural products are of great interest due to their natural diversity as well as leading development of desired therapeutic agents (Khan et al., 2016).

*Quercus incana* is a tree belonging to genus *Quercus* (oak), of family Fagaceae. There are approximately 900 species of genus *Quercus*, among them only six species are available in Pakistan (Nasir and Ali, 1976). The fruit (acorns) part of *Q. incana* has huge medicinal importance and has been used as astringent in digestive disorders, asthma, while the decoction of the bark is used for the treatment of diarrhea and dysentery (Manan et al., 2007). Fruit of *Q. incana* has analgesic effect and has been used for gastrointestinal activity (Sarwar et al., 2013). The leaves of *Q. incana* are known to possess antioxidant nature. These have also found to be effective against certain fungal as well as bacterial strains (Sarwar et al., 2015). Phytochemical study of *Q. incana* revealed the presence of phenolic compounds (Sadanandam and Christopher, 2010), condensed tannins (Jothimanivannan et al., 2010), proanthocyanidins (Kocyigit et al., 2010), and flavonoids (Thaina et al., 2009). Literature showed that the polar fractions from different parts of the genus *Quercus* possess antibacterial activities indicating their ethno-pharmacological use (Berahou et al., 2007).

In the current study, we are reporting the isolation and structure elucidation of new aromatic acid and aromatic alcohol and their anxiolytic- and antidepressant-like effects in mouse models of anxiety and depression.

## Materials and Methods

### Chemicals and Drugs

The analytical grade solvents, flumazenil, imipramine, and the diazepam were purchased from Sigma (St. Louis, MO, United States).

### Animals

Swiss albino male mice 25–30 g were obtained from the National Institute of Health (NIH), Islamabad, Pakistan and maintained in the animal house of the Department of Pharmacy, University of Malakand. The tested animals were acclimatized for a week prior the initiation of research work. Animals were housed in the Department of Pharmacy, University of Malakand’s animal house with fresh water and standard food available *ad libitum*. The animals were maintained at 12 h light and dark cycle and with room temperature maintained at 22–25°C in the animal house. Experiments were conducted in accordance with the accepted guidelines of animal (Scientific Procedures) Act UK 1986. All drugs were dissolved in vehicle consisting of 95% saline, 4% DMSO, 1% Tween-20, and experiments were performed during day time (9 AM to 2 PM). The intraperitoneal administration of all treatments was carried out as 10 ml/kg body weight of mice.

### Plant Extraction and Fractionation

**FIGURE 1:**
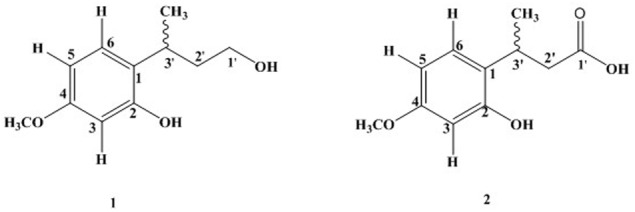
Structure of compounds [**1** and **2**].

*Quercus incana* leaves collection, extraction, and fractionation were done as reported in our previous study (Sarwar et al., 2015). Phytochemical analysis of ethyl acetate fraction through repeated column chromatography resulted in the isolation of two new compounds [**1** and **2**] as shown in **Figure 1**.

### Experimental Procedures

Isolation and purification of compounds were performed by the help of column chromatography using silica gel (70–230 mesh and 230–400 E-Merck) as stationary phase. For TLC analysis, pre-coated (silica gel 60 F_254_) plates were used. Also, IR and HR–EI–MS analysis of pure compounds were performed on UV-240 spectrophotometer JASCO-320-A and double-focusing Varian MAT-312 spectrometer, respectively. The NMR spectral analysis (^1^H and ^13^C) was done by using Bruker AMX-300 spectrometer.

### Pharmacological Experiments

#### Acute Toxicity Studies

The acute toxicity studies were performed according to the method described previously (Karim et al., 2015). Swiss albino male mice were divided into five groups with four mice in each group. Animals were deprived from food overnight but had access to water. After 12 h, animals in groups I and II were treated with compound **1** at the doses of 100 mg/kg, i.p. and 200 mg/kg, i.p. Similarly, groups III and IV received compound **2** at the dose level of 100 and 200 mg/kg, i.p. Animals in the control group received vehicle (10 ml/kg i.p.). Animals were keenly observed for any signs of toxicity for 6 h and then monitored for the next 24 h for changes in behaviors including sedation, convulsions, grooming, hyperactivity, respiratory arrest, increased or decreased motor activity, and mortality.

#### Assessment of Anxiolytic-Like Effect in Elevated Plus-Maze Test

Two open arms in EPM apparatus are connected with two closed arms by an open central platform. The assembly was lifted up from ground level at a height of 40 cm. An elevated edge, the height of which was 3 mm with a total thickness of 1 mm, encircled the open arms of the apparatus. The mice were distributed into 11 groups. Group I received vehicle (10 ml/kg) while diazepam (1 mg/kg, i.p.) was injected as reference to group II. The test compound **1** (1, 10, and 30 mg/kg, i.p.) was selected for treatment of groups III–V while test compound **2** (1, 10, and 30 mg/kg, i.p.) for groups VI–VIII.

After 20 min, animals were placed one by one in the central platform of the EPM facing an open arm and were allowed to explore the EPM for 5 min. An arm entry was defined when all four paws of the mouse were inside the arm. All sessions were recorded with a digital video camera positioned above the EPM. The behavioral parameters including the number of open-arm entries and time spent in open arms were noted at the end of the test from observing the recorded videos. The % open entries and the % time spent in the open arms were considered as a measure of state of anxiety. To evaluate the involvement of GABAergic system in the anxiolytic-like effects of these compounds, mice were pre-treated with either vehicle, flumazenil (2.5 mg/kg, i.p.), or PTZ (10 mg/kg, i.p.) before administration of compounds **1** and **2** or diazepam.

In order to avoid any cues associated with odor, the EPM was cleaned thoroughly with 70% ethanol after each session.

#### Assessment of Anxiolytic-Like Effect in Light/Dark Box Test

The light–dark box (LDB) test apparatus used in this study has been described previously (Karim et al., 2015). Briefly, it consists of a wooden box with 44 cm length, 21 cm width and height each. One-third of this box comprises small compartment whereas two-third of the box makes a large and illuminated compartment. The partition of the box has been done by a board made of wood possessing a 7 × 7 cm opening in the center linking both compartments. Inside walls of the small compartment was painted with black color whereas large compartment was painted white. The portion of the box comprising small compartment was covered with a lid made of wood whereas large compartment was covered with Plexiglas. The large compartment was getting light by a 60-watt bulb which was placed at a height of 30 cm above the test apparatus. A total eight groups of animals were created with six members in each group. Groups I and II were given vehicle (10 ml/kg) and diazepam (01 mg/kg, i.p.), respectively. Test compound **1** (01, 10, and 30 mg/kg, i.p.) was administered to Groups III–V and test compound **2** (01, 10, and 30 mg/kg, i.p.) to groups VI–VIII. Treated mice were given a total time of 20 min and were later placed one by one in illuminated compartment. It was assured that each animal faces the opening in the wooden board away from the dark section. For 5 min, the animal’s behavior was recorded by video camera which was placed at a height of 1 m above the apparatus. The total time spent in illuminated section along with total number of times the animal passes between the two sections were the two parameters noted from the video records as a measure of anxiety. An increased exploration of the lit section and increased transitions between the two compartments of the box are associated with an anti-anxiety effect (Belzung et al., 1987; Bourin and Hascoët, 2003).

#### Antidepressant-Like Effect in the FST

The forced swim test (FST) was also conducted as described previously with minor modifications (Porsolt et al., 1978). The test is conducted in two phases. The pretest is conducted 24 h before the actual test. Imipramine (60 mg/kg) was used as reference drug. In pretest, mice were individually placed in transparent glass tanks (height × width = 45 cm × 18 cm) which were filled up to 25 cm with water and maintained at 25°C. Animals were allowed to swim for 15 min followed by their removal, drying by using a towel, and placement back in their respective cages. After 24 h, vehicle, imipramine, and compound **1** or compound **2** were administered intraperitoneally to the test animals. After 1 h of the compounds administration, the mice were subjected to similar experimental conditions as pretest session and the immobility duration was recorded for 5 min (Porsolt et al., 1978).

#### Antidepressant-Like Effect in the TST

This test was carried out as described earlier (Steru et al., 1985). Mice were administered with vehicle or imipramine (60 mg/kg) or compounds **1** or **2** at the dose level of 1, 10, and 30 mg/kg, i.p. Mice were suspended by their tails and the duration of immobility was recorded for a period of 6 min as described by Steru et al. (1985). The complete motionless suspension of test animals was referred to as a condition of immobility.

#### Approval from Research Ethics Committee

It is certified that the Departmental Research Ethics Committee (DREC) has reviewed the National Research Program for Universities (NRPU) research grants application of the project entitled “Anxiolytic and Antidepressant Activities of Selected Natural product (Glycosides and Flavonoids).” The principal investigator of the project is Dr. Nasiara Karim, Assistant Professor, Department of Pharmacy, University of Malakand.

The committee approves (DAEC/Pharm/2017/01) the study to be conducted in the present form and expects to be informed about any revision in the protocol and subject/patient information/informed consent (where applicable).

### Statistical Data Analysis

The data were expressed as mean ± standard error of the mean (SEM). Data were analyzed by one-way analysis of variance (ANOVA) followed by Dunnett’s *post hoc* test using GraphPad Prism version 5.0. The analyzed values were statistically significant when *P* < 0.05.

## Results

### Column Chromatography

Fraction obtained from ethyl acetate was taken up further to perform column chromatography. Stationary bed used for chromatography was silica gel and *n*-hexane was used as mobile phase, with a gradient of ethyl acetate up to 100% followed by methanol. It resulted in four fractions (fractions A–D). Further, fraction B was loaded on silica gel (flash silica 230-mesh) and eluted with EtOAc: *n*-hexane (70:30) to get compound **1** while compound **2** was purified at EtOAc: *n*-hexane (85:15), respectively.

Compound **1** was new rare class of aromatic alcohol isolated as colorless oil from ethyl acetate fraction of *Q. incana*. The structure was mainly elucidated by ^1^H-NMR, ^13^C-NMR, high resolution mass spectrometry, and supported by 2D-NMR techniques. The molecular ion peak of compound **1** appeared at *m/z* 196 [M]^+^ both in HR-EI-MS and EI-MS spectra suggested molecular formula C_11_H_16_O_3_, consistent with Δ^4^ degree of unsaturation. In addition with its molecular ion peak, it showed characteristic fragments at *m/z* 181, 151, 137, and 91. The HR-EIMS gave exact mass of compound **1** at *m/z* 196.1006 (calcd. 196.1009 for C_11_H_16_O_3_). The IR spectrum showed an absorption peak at 3383 cm^-1^ for the hydroxyl group. The ^1^H-NMR spectrum revealed signals for three aromatic protons at δ_H_ 6.44 (1H, d, *J* = 2.4 Hz, H-3), δ_H_ 6.50 (1H, dd, *J* = 8.1, 2.4 Hz, H-5), and δ_H_ 7.06 (1H, d, *J* = 8.1 Hz, H-6), while the methoxy group at aromatic ring appeared at δ_H_ 3.77 (3H, s, OCH_3_). The only methyl group appeared at δ_H_ 1.31 (3H, d, *J* = 7.1Hz, CH_3_), while the methine signal centered at δ_H_ 3.20 (1H, m, H-3′) as shown in **Table 1**. Similarly, the methylene signal bearing hydroxyl group resonated at δ_H_ 3.39 (1H, m, H-1′), δ_H_ 3.68 (1H, m, H-1′) and the other methylene signal appeared at δ_H_ 1.53 (1H, m, H-2′), δ_H_ 2.01 (1H, m, H-2′). Methylene proton (H-5) showed *ortho* coupling with H-6 at δ_H_ 7.06 (1H, d, *J* = 8.1 Hz) and *meta* coupling with H-3 at δ_H_ 6.44 (1H, d, *J* = 2.4 Hz) characteristic of aromatic ring (Lal et al., 1987).

**Table 1:** ^1^H-NMR (CDCl_3_, 300 MHz) data of compounds [**1** and **2**] in ppm, *J* in Hz.

Position	1	2
1	-	-
2	-	-
3	6.44 (d, *J* = 2.4 Hz)	6.45 (d, *J* = 2.5 Hz)
4	-	-
5	6.50 (dd, *J* = 8.1, 2.4 Hz)	6.52 (dd, *J* = 8.1, 2.5 Hz)
6	7.06 (d, *J* = 8.1 Hz)	7.06 (d, *J* = 8.1Hz)
7	-	-
8	-	-
9	-	-
10	-	-
1′	3.39, m	-
	3.68, m	
2′	1.53 m	2.90, m
	2.01, m	3.08, m
3′	3.20, m	3.29, m
7-CH_3_	1.31 (d, *J* = 7.1)	1.30 (d, *J* = 7.8)
8-OCH_3_	3.77, s	3.74, s

**FIGURE 2:**
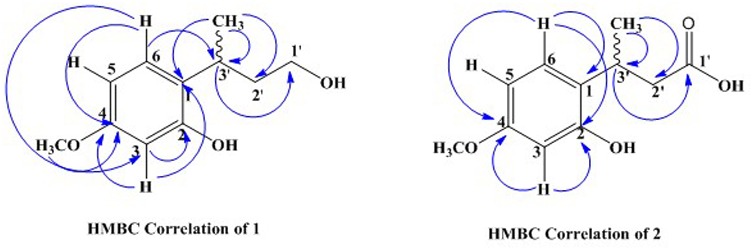
Important HMBC correlation of compounds [**1** and **2**].

The ^13^C-NMR spectra showed the presence of 11 carbon atom, including one methyl, one methoxy group, two methylene, four methine, and three quaternary carbons. The ^13^C-NMR revealed the presence of aromatic carbon signal at δ_C_ 124 (C-1), 155.1 (C-2), 102 (C-3), 158.6 (C-4), 106.9 (C-5), and 127.2 (C-6). Similarly, the signal for methoxy carbon appeared at δ_C_ 55.8, while methyl group resonated at δ_C_ 21.1. The downfield methylene appeared at δ_C_ 60.9 (C-1′) while other methylene signal was observed at δ_C_ 40.7 (C-2′). The HMBC spectrum confirmed the position of methoxy group at aromatic ring having HMBC correlation with C-4, C-5, and C-3. Similarly, the other data derived from ^1^H-NMR and ^13^C-NMR were supported by HMBC connectivity. The placement of the side chain at C-1 of the aromatic ring was supported by H-H-COSY and HMBC correlation (**Figure 2**). The HMBC correlation of the methine proton (δ_H_ 3.20) showed strong connectivity with aromatic carbon C-1, C-2, and C-6, and this proton was further correlated to CH_3_, C-1′, and C-2′. Likewise the placement of CH_3_ group at C-3′ was also suggested by HMBC which in turn showed correlation with C-2′, C-3′, and aromatic C-1. The structure proposed from spectral data for compound **1** was 2-(4-hydroxybutan-2-yl)-5-methoxyphenol.

The isolated compound **2** (colorless oil) was an aromatic acid. The EI–MS data which showed molecular ion peak at *m/z* 210 and HR–EI–MS gave the exact mass of compound **2** as *m/z* 210.0899 with molecular formula C_11_H_14_O_4_, having Δ^5^ degree of unsaturation. The compound **2** showed some characteristic mass fragments at *m/*z 192, 164, and 117. The IR absorption showed the presence of hydroxyl group signal at 3502 cm^-1^ and carboxyl group at 1721 cm^-1^. The ^1^H-NMR spectrum showed the presence of aromatic proton at position H-5 (δ_H_ 6.52, 1H, dd, *J* = 8.1, 2.5 Hz) having *ortho* and *meta* coupling with H-6 (δ_H_ 7.06, 1H, d, *J* = 8.1 Hz) and H-4 (δ_H_ 6.45, 1H, d, *J* = 2.5 Hz), respectively, quite identical to compound **2** (Lal et al., 1987). In addition, the methyl group resonated at δ_H_ 1.30 (3H, d, *J* = 7.8Hz, CH_3_) and methine signal centered at δ_H_ 3.29 (1H, m, H-3′). The methylene signal appeared at δ_H_ 3.40 (1H, m, H-2′), δ_H_ 3.69 (1H, m, H-2′). The methoxy group in compound **2** at aromatic ring was resonated at δ_H_ 3.74 (3H, s, OCH_3_) while the methylene signal appeared at δ_H_ 2.90 (1H, m, H-2′), δ_H_ 3.08 (1H, m, H-2′). The ^13^C-NMR spectra showed the presence of 11 carbon atoms, including one methyl, one methoxy group, one methylene, three methine, and four quaternary carbons (as shown in **Table 2**).

**Table 2:** ^13^C-NMR (CDCl_3_, 75 MHz) data of compounds [**1** and **2**] in ppm.

Position	1	2
1	124.1	124.9
2	155.1	155.1
3	102.1	106.1
4	158.6	159.7
5	106.9	106.7
6	127.2	126.5
7	-	-
8	-	-
9	-	-
10	-	-
1′	60.9	178.4
2′	40.7	52.9
3′	26.3	26.1
7-CH_3_	21.1	20.9
8-OCH_3_	55.8	55.8

The carbon spectrum showed aromatic signals at δ_C_ 124.9 (C-1), 155.1 (C-2), 106.1 (C-3), 159.7 (C-4), 106.7 (C-5), and 126.5 (C-6). Similarly, the signal for methoxy group appeared at δ_C_ 55.8, while methyl group resonated at 20.9 and downfield methylene appeared at δ_C_ 52.9 (C-2′). The HMBC correlations of compound **2** confirmed the position of methoxy group at aromatic ring having interactions with C-4, C-5, and C-3 (**Figure 2** and **Tables 1**, **2**). All other data derived from ^1^H-NMR and ^13^C-NMR were supported by HMBC connectivity. The HMBC correlation of the methine proton δ_H_ 3.29 showed strong connectivity with C-1, C-2 and C-6, C-1′, C-2′, and CH_3_. Likewise, position of CH_3_ at C-3′ is also suggested through HMBC which in turn presents correlation with C-2′, C-3′, as well as aromatic C-1. Finally, the structure of compound **2** was found as 3-(2-hydroxy-4-methoxyphenyl) butanoic acid.

### Characterization of Compound **1**

Colorless oil: IR (KBr) *υ*_max_ 3383 cm^-1^. EI–MS *m*/*z:* (rel. int.) 196 [M]^+^ (35), 181 (20), 151 (100), 137 (25), 91 (37). HR–EI–MS: *m*/*z* 196.1006 (calcd. 196.1099 for C_11_H_16_O_3_). ^1^H-NMR (CDCl_3_, 300 MHz): δ_H_ 6.44 (1H, d, *J* = 2.4 Hz, H-3), 6.50 (1H, dd, *J* = 8.1, 2.4 Hz, H-5), 7.06 (1H, d, *J* = 8.1 Hz, H-6), 3.20 (1H, m, H-1′), 1.53 (1H, m, H-2′), 2.01 (1H, m, H-2′), 3.68 (1H, m, H-3′), 3.39 (1H, m, H-3′), 1.31 (CH_3_, d, *J* = 7.1 Hz, H-7), 3.77 (OCH_3_, s). ^13^C-NMR (CDCl_3_, 75 MHz): δ_C_ 124.1 (C-1), 155.1 (C-2), 102.4 (C-3), 158.6 (C-4), 106.9 (C-5), 127.2 (C-6), 60.9 (C-1′), 40.7 (C-2′), 26.3 (C-3′), 21.1 (CH_3_), 55.8 (OCH_3_).

### Characterization of Compound **2**

Colorless oil: IR (KBr) *υ*_max_ 3502, 1761 cm^-1^. EI–MS *m*/*z:* (rel. int.) 210 [M]^+^ (11), 192 (40), 164 (33), 117 (65). HR–EI–MS: *m*/*z* 210.0899 (calcd. 210.0892 for C_11_H_14_O_4_). ^1^H-NMR (CDCl_3_, 300 MHz): δ_H_ 6.45 (1H, d, *J* = 2.5 Hz, H-3), 6.52 (1H, dd, *J* = 8.1, 2.5 Hz, H-5), 7.06 (1H, d, *J* = 8.1 Hz, H-6), 1.30 (3H, d, *J* = 7.8 Hz, H-7), 3.74 (3H, s, H-8), 2.90, 3.08 (2H, m, H-2′), 3.29 (1H, m, H-3′), ^13^C-NMR (CDCl_3_, 75 MHz): δ_C_ 124.9 (C-1), 155.1 (C-2), 106.1 (C-3), 159.7 (C-4), 106.7 (C-5), 126.5 (C-6), 178.4 (C-1′), 52.9 (C-2′), 26.1 (C-3′), 20.9 (CH_3_), 55.8 (OCH_3_).

### Acute Toxicity

No pronounced changes in the behavior of animals were evident by compounds [**1** and **2**] at dosage levels of 100 and 200 mg/kg. There had been observed no effect on sedation, respiration, convulsions and grooming, or muscle activity.

### Anxiolytic-Like Effects in EPM Test

**FIGURE 3:**
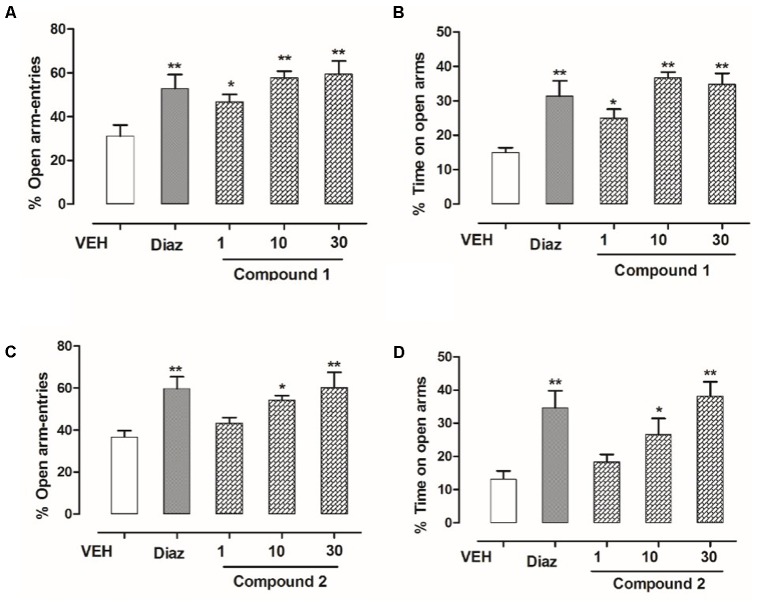
Effect of compounds **1** and **2** and diazepam on the behavior of mice in the EPM. **(A)** % open-arm entries, **(B)** % time spent in open arms registered over a session of 5 min, after 20 min of an i.p. injection of compound **1** (1, 10, and 30 mg/kg), diazepam (1 mg/kg), or vehicle. **(C)** % open-arm entries **(D)** % time spent in open arms registered over a session of 5 min, after 20 min of an i.p. injection of compound **2** (1, 10, and 30 mg/kg), diazepam (1 mg/kg), or vehicle. Column represents mean ± SEM (*n* = 6/group). ^∗^*P* < 0.05, ^∗∗^*P* < 0.01, compared with vehicle group using one-way ANOVA followed by Dunnett’s test.

The effect of 3′-MeO6MF or diazepam in the behavior of mice in elevated plus maze (EPM) is given in **Figure 3**. Both diazepam and compounds **1** and **2** significantly reduced the anxiety in mice. Compound **1** at dose levels of 01, 10, and 30 mg/kg, i.p., effectively resulted in an increase of % open-arm entries as well as % time spent in open arms of the EPM (^∗^*P* < 0.05, ^∗^*P* < 0.01, respectively, *n* = 6, one-way ANOVA followed by Dunnett’s test). As shown in **Figure 3**, compound **2** at dose levels of 10 and 30 mg/kg also showed pronounced increase in open-arm entries and % increase in time spent within open arm (^∗^*P* < 0.05, ^∗^*P* < 0.01, respectively, *n* = 6, one-way ANOVA followed by Dunnett’s test). The standard reference drug diazepam at 1 mg/kg, i.p., also increased % open-arm entries and % time spent in open arms of the EPM (^∗∗^*P* < 0.01, *n* = 6, one-way ANOVA followed by Dunnett’s test).

### Effect of Flumazenil on the Anxiolytic-Like Activity of Compounds **1** and **2** in the EPM

**FIGURE 4:**
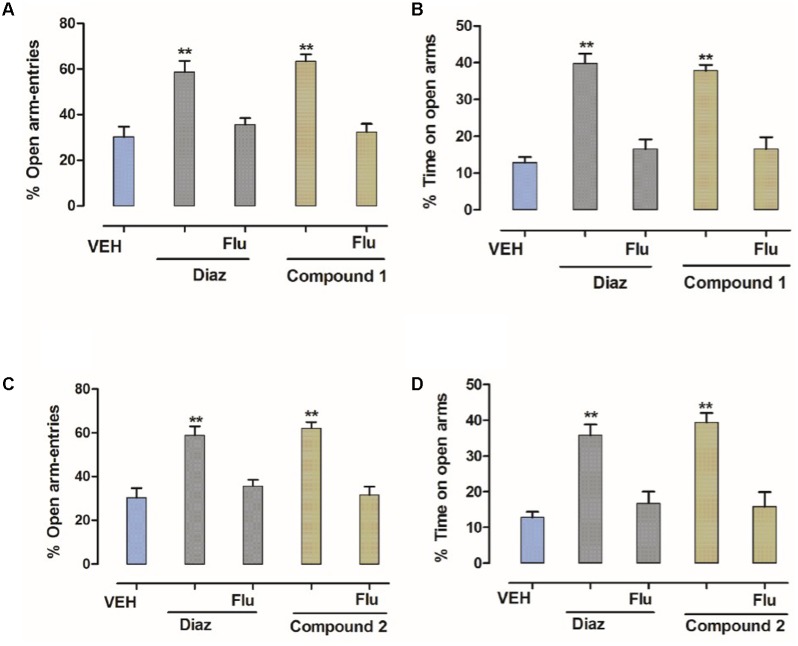
Effect of flumazenil on the anxiolytic activity of compounds **1** and **2** in the EPM. **(A)** % entries in open arms, **(B)** % time spent in open arms registered over a session of 5 min, after 20 min of an i.p. injection of vehicle, diazepam (1 mg/kg), and compound **1** (10 mg/kg), **(C)** % entries in open arms, **(D)** % time spent in open arms registered over a session of 5 min, after 20 min of an i.p. injection of vehicle, diazepam (1 mg/kg), and compound **2** (10 mg/kg) in the presence of flumazenil (2.5 mg/kg). Columns express mean ± SEM (*n* = 6/group), *n* = 6 per group. ^∗∗^*P* < 0.01, compared with vehicle control group using one-way ANOVA post-Dunnett’s test. Co-administration of flumazenil (2.5 mg/kg) blocked the anti-anxiety action of compounds **1** and **2** (10 mg/kg showing the anxiolytic effect is mediated via the benzodiazepine site.

In order to identify the involvement of BDZ-binding site, flumazenil was used to antagonize the effects of compounds **1** and **2**. As shown in **Figure 4**, flumazenil (2.5 mg/kg) completely abolished the increases in the number of open-arm entries and % increase in time spent in open arms by compounds **1** and **2**. This effect was similar to that of diazepam indicating that compounds **1** and **2** (10 mg/kg) were mediating its anxiolytic-like effect *via* the BDZ receptors (**Figure 4**).

### Anxiolytic-Like Effects of Compounds 1 and 2 in Light Dark/Dark Box Test

**FIGURE 5:**
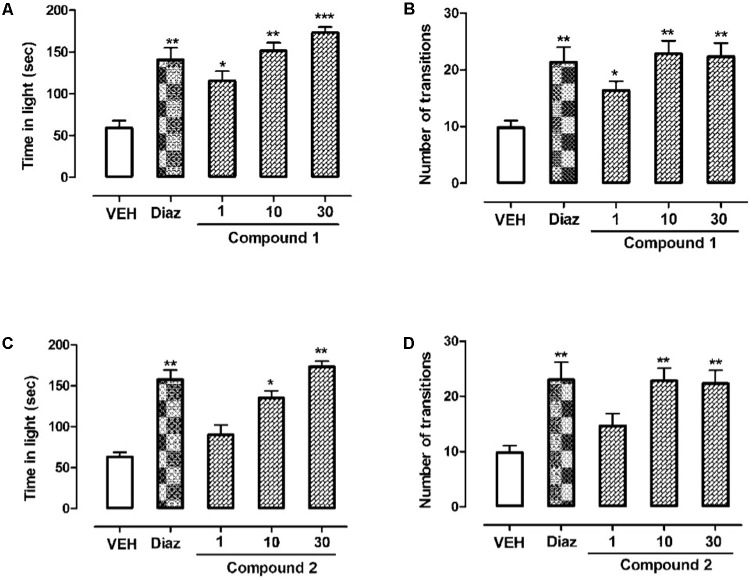
Effect of compounds **1** and **2** and diazepam on the behavior of mice in the light–dark box test. **(A)** Time spent in lit area, **(B)** number of transitions recorded over a session of 5 min, after 20 min of an i.p. injection of compound **1** (1, 10, and 30 mg/kg), diazepam (1 mg/kg), or vehicle. **(C)** Time spent in lit area, **(D)** number of transitions recorded over a session of 5 min, after 20 min of an i.p. injection of compound **2** (1, 10, and 30 mg/kg), diazepam (1 mg/kg), or vehicle. Values express mean ± SEM (*n* = 6/group). ^∗^*P* < 0.05, ^∗∗^*P* < 0.01, and ^∗∗∗^*P* < 0.001 compared with vehicle group using one-way ANOVA followed by Dunnett’s test.

The behavioral effects of diazepam, compounds **1** and **2**, or vehicle in the light–dark box (LDB) test are shown in **Figure 5**. Outcomes from ANOVA analysis revealed that compound **1**, at the dose levels (10 and 30 mg/kg, i.p), has not only effectively increased time spent in light compartment but also increased the transitions across two compartments (^∗^*P* < 0.05, ^∗∗^*P* < 0.01, respectively, *n* = 6, one-way ANOVA followed by Dunnett’s test). Similar results were obtained for compound **2** which also significantly enhanced time spent in light compartment as well as number of transitions across the two compartments (^∗^*P* < 0.05, ^∗∗^*P* < 0.01, respectively, *n* = 6, one-way ANOVA followed by Dunnett’s test) at dose levels of 10 and 30 mg/kg. The diazepam which was taken as reference drug (1 mg/kg, i.p.) also significantly (^∗∗^*P* < 0.01; n = 6, one-way ANOVA followed by Dunnett’s test) enhanced time spent in light area of the light–dark box showing anti-anxiety potential, which confirmed the validity of paradigm.

### Antidepressant-Like Effects in Forced Swimming Test

The effects of compounds **1** and **2** in mice FST were summarized in **Table 3**. Intraperitoneal administration of compounds **1** and **2** at doses of 10 and 30 mg/kg significantly decreased the immobility time in the FST compared to the control (vehicle) (^∗^*P* < 0.05; ^∗∗^*P* < 0.01; one-way ANOVA, followed by Dunnett’s test). Imipramine was used as a reference drug which also significantly decreased the immobility time in comparison with vehicle at a dose of 60 mg/kg (^∗∗∗^*P* < 0.001; one-way ANOVA followed by Dunnett’s test).

**Table 3:** Effect of compounds **1** and **2** on immobility period (seconds) of mice using forced swimming test.

Treatment	Dose (mg/kg)	Immobility time (seconds)
Control	-	170.5 ± 8.5
Compound **1**	1	168.4 ± 6.5
	10	120.5 ± 5.5*
	30	85.3 ± 7.2**
Compound **2**	1	169.4 ± 4.4
	10	145.3 ± 4.5*
	30	105.5 ± 10.3**
Imipramine	60	75.5 ± 9.6***

*All values are expressed in mean ± SEM (*n* = 6). ^∗^*P* < 0.05, ^∗∗^*P* < 0.01, ^∗∗∗^*P* < 0.001 compared to the vehicle group. Difference between groups was analyzed by ANOVA (one-way ANOVA followed by Dunnett’s test)*.

### Antidepressant-Like Effects in Tail Suspension Test

The effects of compounds **1** and **2** in the tail suspension test (TST) were shown in **Table 4**. Intraperitoneal administration of the reference drug, imipramine at the dose of 60 mg/kg, significantly decreased the immobility time compared to the vehicle (^∗∗∗^*P* < 0.001; one-way ANOVA, followed by Dunnett’s test). Compounds **1** and **2** at the doses of 10 and 30 mg/kg also caused a significant decrease in the immobility time compared to the vehicle control group (^∗^*P* < 0.05; ^∗∗^*P* < 0.01; one-way ANOVA, followed by Dunnett’s test).

**Table 4:** Effect of compounds 1 and 2 on immobility period (seconds) of mice using tail suspension test.

Treatment	Dose (mg/kg)	Immobility time (seconds)
Control	-	165.6 ± 12.5
Compound 1	1	159.4 ± 8.5
	10	130.5 ± 5.5*
	30	90.5 ± 4.2**
Compound 2	1	161.4 ± 8.4
	10	141.3 ± 6.5*
	30	96.5 ± 7.5**
Imipramine	60	80.5 ± 9.5***

*All values are expressed in mean ± SEM (*n* = 6). ^∗^*P* < 0.05, ^∗∗^*P* < 0.01, ^∗∗∗^*P* < 0.001 compared to the vehicle group. Difference between groups was analyzed by ANOVA (one-way ANOVA followed by Dunnett’s test)*.

## Discussion

Anxiety and depression are the most prevalent health problems among other mood disorders worldwide. In this study, the anxiolytic- and antidepressant-like effects of compounds **1** and **2** had been studied in different animal models of anxiety and depression. In this study, compounds **1** and **2** exerted significant anxiolytic effects in both EPM and light dark tests (LDTs). In the EPM, compound **1** dose dependently increased the exploratory behavior of mice by increasing both the % open-arm entries and time spent on open arms (^∗^*P* < 0.05, ^∗∗^*P* < 0.01, ^∗∗∗^*P* < 0.001; *n* = 6). Similarly, compound **2** also significantly increased the % open-arm entries and time spent on open arms at the dose level of 10 and 30 mg/kg indicating anxiolytic-like effects (^∗^*P* < 0.05, ^∗∗^*P* < 0.01; *n* = 6). Co-administration flumazenil (2.5 mg/kg) with compounds **1** and **2** (10 mg/kg) completely abolished the anxiolytic-like activity of compounds **1** and **2** indicating that the BZP site of GABAergic system was involved in the anxiolytic-like activity. In LDB test, the ANOVA analysis demonstrates that compound **1** at the dose level of 1–30 mg/kg exerted significant anxiolytic-like effect (^∗^*P* < 0.05, ^∗∗^*P* < 0.01, ^∗∗∗^*P* < 0.001; *n* = 6). Similarly, compound **2** showed significant anxiolytic activity at the dose level of 10 and 30 mg/kg (^∗^*P* < 0.05, ^∗∗^*P* < 0.01; *n* = 6). The anxiolytic-like effect of both compounds at 30 mg/kg was comparable to the standard reference drug diazepam in both EPM and LDB tests.

The EPM and LDTs were the most important behavioral assays for the assessment of anxiolytic-like effect. Several studies have reported that GABAergic neurotransmission was involved in expression etiology and therapy of anxiety disorders (Chakraborty et al., 2010). The sensitivity of EPM to the effect of both anti-anxiety and anxiogenic drug acting at the GABA_A_ BZP complex was very high (Nic Dhonnchadha et al., 2003). In EPM, normal animals were normally chosen to spend much of their allowed time in the closed arms. This orientation seems to reflect a distaste toward open arms that was produced by the anxiety of the open spaces. Drug like diazepam that enhances exploration on open arm are believed as anxiolytic and the opposite holds right for anxiogenics.

Administration of compounds **1** and **2** prior to test significantly decreased the total immobility time compared to the vehicle control. Similar results were obtained in TST, another primary screening test for detecting antidepressant substances. The TST also induces a state of despair in animals similar to that in FST (Steru et al., 1985). Both compounds [**1** and **2**] dose dependently decreased the immobility time in TST. The reduction in immobility time in both TST and FST was dose dependent with no effect at 1 mg/kg.

The tail suspension and forced swimming tests are the most validated animal models to evaluate substances with putative antidepressant-like effects (Porsolt et al., 1977; Steru et al., 1985). In FST, when rodents are forced to swim in a confined, they tend to become immobile after an initial period of struggling. This inescapable stressful situation is evaluated by assessing different behavioral parameters (Porsolt et al., 1978).

Several neurotransmitters have been implicated in the pathophysiology of depressive disorders including GABA, serotonin, noradrenalin, and dopamine (Dailly et al., 2004; Abdelhalim et al., 2015). Thus, depression has been believed to be due to deficiency of one or another of these neurotransmitters (Willner et al., 2005; Luscher et al., 2011). Furthermore, in the present study, flumazenil was able to antagonize the anxiolytic-like effect of compounds **1** and **2** in the EPM and LDB test suggests the involvement of these biogenic amines in the antidepressant-like effects of these compounds.

## Conclusion

Compounds **1** and **2** exerted anxiolytic and antidepressant-like effects in classical mouse models of anxiety and depression. Additionally the results indicated that the anxiolytic-like effect may involve the BZP site of GABA-A receptors. Thus, this study provides valuable preliminary data on the anxiolytic and antidepressant-like effects of compounds **1** and **2** isolated from *Q. incana*. However, further studies are required to elucidate the antidepressant-like mechanisms and to conduct further preclinical and clinical studies of these compounds.

## Author Contributions

AK and UF conceived and designed the study. RS and SN performed the isolation and SB helped in the structure elucidation. NK and IK performed *in vivo* studies. HK and AA analyzed the data. UF, AK, and AA wrote the manuscript with inputs and comments from all co-authors. All authors read and approved the final version of the manuscript.

## Conflict of Interest Statement

The authors declare that the research was conducted in the absence of any commercial or financial relationships that could be construed as a potential conflict of interest.
